# Completion of perioperative chemotherapy and tumor regression grade as independent predictors of one-year survival after total gastrectomy for gastric cancer: a retrospective cohort study

**DOI:** 10.1186/s12957-026-04364-w

**Published:** 2026-04-25

**Authors:** Mazen Aldarwish, Jens P. Hoelzen, Nader El-Sourani, Carsten Szardenings, Ahmed Abdelsamad, Dhruvajyoti Roy, Martina Holstein, Ann-Kathrin Eichelmann, Jennifer Merten, Simon Kammer, Dmitry Shapiro, Wolfgang Hartmann, Eva Wardelmann, Andreas Pascher, Mazen A. Juratli

**Affiliations:** 1 Department of General, Visceral and Transplant Surgery, University Hospital Muenster, Albert-Schweitzer-Campus 1, Geb. W1, Muenster, 48149 Germany; 2 Otto Creutzfeldt Center for Cognitive and Behavioral Neuroscience, University of Muenster, Muenster, 48149 Germany; 3 Department of Surgery II, University of Witten/Herdecke, Witten, Germany; 4 Department of Breast Surgical Oncology, University Hospital of Texas, MD Anderson Cancer Center, TX Houston, United States; 5 Gerhard-Domagk-Institute of Pathology, University Hospital Muenster, Muenster, Germany

**Keywords:** Gastric cancer, Survival, Perioperative chemotherapy, Tumor regression grade, Lymph node ratio

## Abstract

**Background:**

Gastric cancer remains a major global health burden, with persistently high mortality rates despite advances in multimodal treatment. Total gastrectomy (TG) constitutes a cornerstone of curative therapy; however, the factors governing early postoperative survival remain incompletely characterized. This study aimed to identify clinical and pathological predictors of 1-year overall survival (OS) following curative-intent TG, with particular emphasis on the oncological treatment strategy and tumor regression grade (TRG).

**Methods:**

We retrospectively analyzed 145 patients who underwent TG between 2012 and 2023, excluding *n* = 4 adjuvant-only cases. To avoid statistical collinearity, multivariable Cox proportional hazards regression was performed in two sequential steps: Model 1 assessed the treatment strategy across the overall cohort (*N* = 145), while Model 2 evaluated TRG exclusively within the neoadjuvant-treated subgroup (*n* = 85). Both models incorporated the lymph node ratio (LNR) and surgical approach, and were adjusted for resection margin status (R-status), comorbidity burden (CCI), and severe postoperative complications (Clavien-Dindo ≥ III). A 60-day landmark analysis was conducted to mitigate immortal time bias.

**Results:**

Completion of the perioperative chemotherapy sequence was independently associated with significantly improved 1-year OS compared to neoadjuvant therapy alone (HR = 0.20; 95% CI, 0.08–0.50; *p* = 0.001). This survival advantage remained highly significant in the 60-day landmark analysis (*p* = 0.004). Notably, 55.3% of patients who initiated neoadjuvant chemotherapy did not proceed to the adjuvant phase, primarily owing to patient refusal or medical contraindications. When evaluated exclusively within the neoadjuvant-treated subgroup, a poorer TRG demonstrated a prognostic trend toward decreased survival (HR = 1.60; 95% CI, 0.98–2.59; *p* = 0.059). Although severe complications (CD ≥ III) occurred in 55.9% of patients, their incidence did not differ significantly across treatment groups (*p* = 0.894) and did not diminish the independent prognostic value of treatment completion. The surgical approach (robotic vs. open) exerted no significant effect on 1-year OS (HR = 0.88; *p* = 0.745).

**Conclusions:**

Completion of the perioperative chemotherapy sequence and a favorable TRG represent two distinct and critical determinants of 1-year survival following TG for gastric cancer. While residual selection bias inherent to retrospective analyses must be acknowledged, the prognostic advantage conferred by treatment completion remains robust after adjustment for surgical morbidity, R-status, and immortal time bias. These findings underscore the prognostic importance of treatment adherence and tumor chemosensitivity, and highlight the need for individualized perioperative management strategies.

**Supplementary Information:**

The online version contains supplementary material available at 10.1186/s12957-026-04364-w.

## Introduction

Gastric cancer poses a major global health challenge, ranking among the most common malignancies and leading causes of cancer-related mortality worldwide, accounting for over 1 million new diagnoses and approximately 770,000 deaths annually [1, 2]. In Germany, it represents a substantial oncological burden, with approximately 14,500 new cases and 8,300 deaths reported each year [3]. Although incidence rates have declined in certain Western nations — partly attributable to effective Helicobacter pylori eradication [4] — the prognosis, particularly for locally advanced or metastatic disease, remains persistently poor [5]. This clinical reality underscores the ongoing need to refine therapeutic approaches and improve patient outcomes [6].

Surgical resection remains the cornerstone of curative treatment for localized gastric cancer [7, 8]. However, a multimodal approach integrating systemic chemotherapy has become the established standard of care for locally advanced, resectable disease (generally stage cT2 or higher, or node-positive) [9]. Perioperative chemotherapy, encompassing both neoadjuvant and adjuvant cycles, represents the preferred strategy in many regions. Its fundamental survival benefit was first demonstrated by the landmark MAGIC trial, which established improved outcomes compared to surgery alone [10]. Subsequently, the FLOT4 trial demonstrated the superiority of the FLOT regimen (docetaxel, oxaliplatin, leucovorin, 5-fluorouracil) over previous standards, establishing it as the current reference regimen for medically fit patients per major clinical guidelines [11]. This perioperative strategy aims to optimize outcomes by downstaging the primary tumor preoperatively and eradicating micrometastatic disease postoperatively.

Despite the demonstrated efficacy of perioperative regimens such as FLOT, considerable variability in real-world patient outcomes persists. Translating clinical trial protocols into routine practice poses substantial challenges, as a significant proportion of patients fail to initiate or complete the full chemotherapy course due to toxicity, severe comorbidities, or disease progression [12]. Consequently, treatment sequences in clinical practice frequently diverge from intended protocols, resulting in patients who receive only neoadjuvant therapy before surgery, or those who undergo primary surgery without preceding systemic treatment. The relative effectiveness of these divergent, real-world treatment sequences compared to the complete perioperative protocol warrants rigorous investigation. This is particularly relevant following major procedures such as total gastrectomy (TG), where substantial surgical morbidity and diminished postoperative physiological reserves often compromise a patient’s ability to complete the intended multimodal sequence.

Moreover, while the prognostic significance of pathological tumor regression (TRG, Becker classification) and the lymph node ratio (LNR) are well established [13–15], their precise contribution across these distinct therapeutic pathways requires further elucidation, particularly when accounting for the biological differences between patients undergoing primary surgery and those receiving neoadjuvant therapy. Additionally, when comparing survival outcomes among different treatment sequences, the potential influence of immortal time bias — a prevalent methodological pitfall in perioperative oncology — must be rigorously addressed to ensure the validity of any findings. Finally, the influence of the surgical approach — specifically robotic-assisted versus conventional open gastrectomy — on oncological outcomes within a multimodal treatment setting remains a subject of ongoing debate.

To address these questions, this retrospective study investigated the association between key clinical variables and 1-year overall survival following curative-intent TG for gastric cancer. Specifically, we evaluated the prognostic impact of the actually administered oncological treatment strategy, pathological TRG, LNR, and surgical approach within a real-world cohort treated over more than a decade. Critically, our methodology incorporates a 60-day landmark analysis to mitigate immortal time bias and adjusts for major clinical confounders, including resection margin status (R-status), baseline physiological reserve (CCI), and severe postoperative complications (CD ≥ III). By examining these multifaceted factors, this study aims to clarify early survival dynamics and contribute to ongoing efforts to optimize and personalize treatment strategies for gastric cancer in routine clinical practice.

## Methods

### Study design and ethical considerations

This study was conducted as a retrospective single-center cohort study, analyzing patients who underwent curative-intent (R0/R1) total gastrectomy (TG) for gastric cancer between January 2012 and October 2023. The study was restricted to total gastrectomy to ensure a homogeneous surgical cohort, as TG is associated with significantly greater perioperative morbidity than subtotal resection, which directly affects the feasibility and tolerability of adjuvant chemotherapy. The study was conducted at the [Department of General, Visceral and Transplantation Surgery, University Hospital Münster, Germany]. The study adhered to the ethical principles outlined in the Declaration of Helsinki (2013 revision) [16]. Ethical approval was obtained from the Ethics Committee of the Medical Association of Westphalia-Lippe and the University of Münster (Reference: 2022-123-f-S). Written informed consent was obtained from all participants prior to data collection and analysis. The study was registered in the German Clinical Trials Register (DRKS) under reference number [DRKS00035827]. This study is reported in accordance with the Strengthening the Reporting of Observational Studies in Epidemiology (STROBE) guidelines.

To mitigate potential selection and immortal time biases inherent in perioperative treatment comparisons, the study incorporated a 60-day landmark analysis coupled with multivariable survival modeling. The 60-day threshold was chosen to ensure that all patients had survived the immediate postoperative recovery period and thus had a realistic clinical opportunity to initiate the adjuvant phase of the perioperative sequence.

### Patient selection and participant flow

**Fig. 1:**
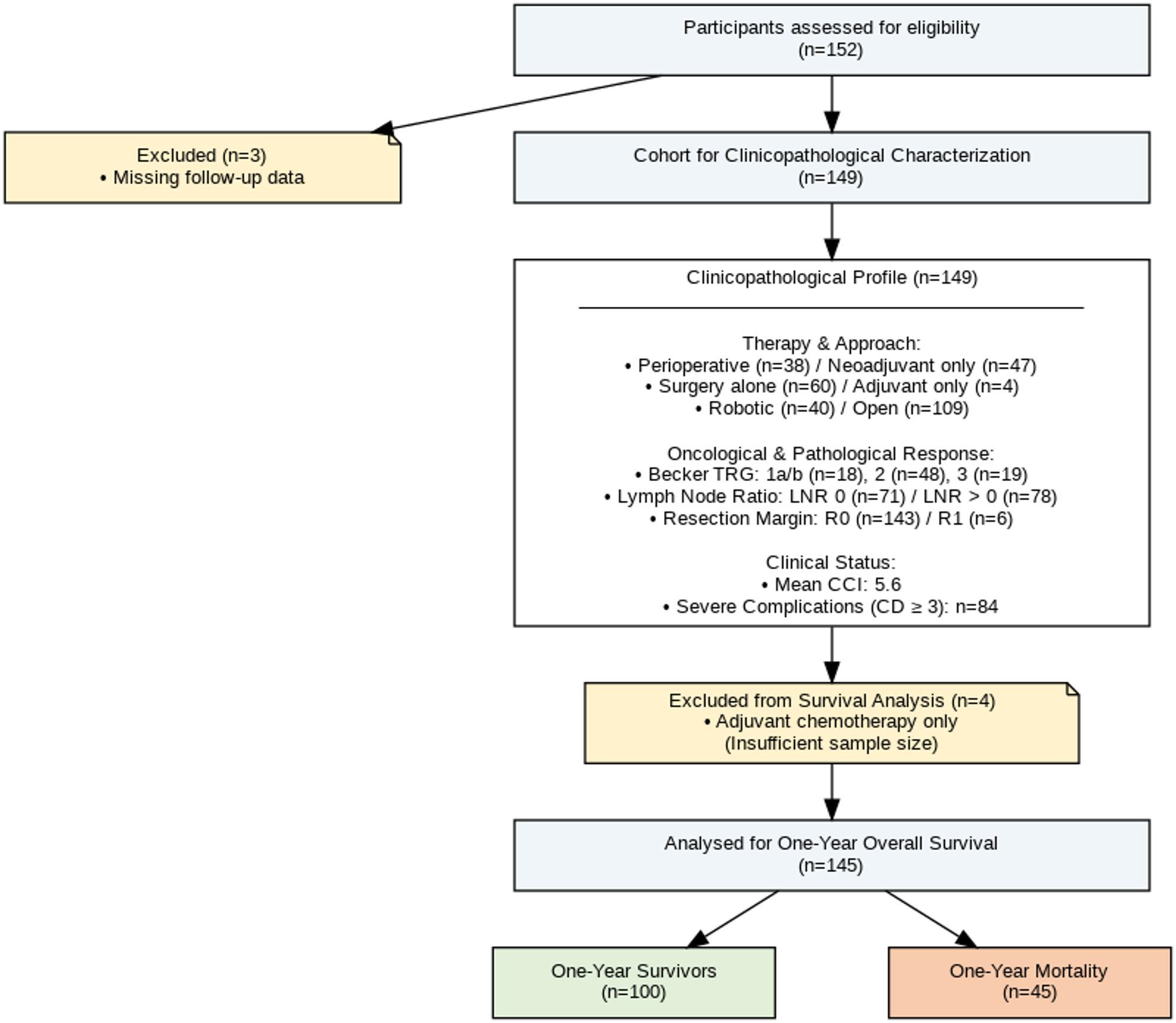
Flow diagram according to STROBE guidelines, depicting participant assessment, exclusion, and inclusion for analysis

The patient selection process is illustrated in the STROBE flow diagram (Fig. 1). Initially, 152 patients undergoing TG were assessed for eligibility. Three patients (1.97%) were excluded due to incomplete follow-up data (following gastrectomy at our institution, they received subsequent oncological care at external facilities). Consequently, a cohort of 149 patients was included for comprehensive clinicopathological characterization.

For the primary survival analysis, patients receiving adjuvant chemotherapy alone were excluded due to the limited sample size of this subgroup (*n* = 4), which precluded meaningful statistical comparison. Thus, the final multivariable survival model was based on the remaining 145 patients. These patients were stratified by oncological treatment strategy (none, neoadjuvant only, or perioperative completion), lymph node ratio (LNR), calculated as the ratio of positive lymph nodes to the total number of harvested nodes, surgical approach (robotic vs. open), and tumor regression grade (Becker classification). Assessment of one-year survival in this analysis cohort revealed that 100 patients were alive and 45 had died within one year of surgery.

### Study population and data collection

Patients undergoing elective, curative-intent (R0/R1) TG for histologically confirmed primary gastric adenocarcinoma during the study period were eligible for inclusion. Exclusion criteria comprised palliative surgery, non-adenocarcinoma histologies, emergency surgery, and incomplete clinical, pathological, or follow-up data precluding primary outcome analysis. Of note, patients classified as postoperative UICC Stage IV represented cases of occult metastatic disease (e.g., positive peritoneal cytology [Cy1] or limited, localized peritoneal nodules [P1]) discovered only intraoperatively. In these cases, the surgical team proceeded with the planned total gastrectomy aiming for macroscopic clearance within a curative-intent multimodal strategy; patients with preoperatively known systemic metastases or those undergoing planned palliative resections were strictly excluded. Eligible patients were identified from a prospectively maintained institutional database. From an initial cohort of 152 patients, three were excluded due to incomplete follow-up data, resulting in a study population of 149 patients for clinicopathological characterization. The patient selection process is detailed in the STROBE flow diagram (Fig. 1).

Comprehensive clinicopathological data were retrospectively collected from electronic health records and pathology reports. Baseline characteristics included age at surgery, sex, body mass index (BMI, kg/m²), and the Charlson Comorbidity Index (CCI) to quantify baseline physiological reserve. Tumor characteristics encompassed primary location and pathological tumor-node-metastasis (pTNM) stage, classified according to the edition of the Union for International Cancer Control (UICC) staging system current at the time of treatment (7th or 8th edition).

The lymph node ratio (LNR), defined as the number of positive lymph nodes divided by the total number of harvested lymph nodes, was calculated for each patient to provide a granular assessment of nodal burden. Treatment information recorded included the actually administered oncological treatment strategy, surgical approach (robotic-assisted vs. conventional open), and microscopic resection margin status (R0 vs. R1). For patients receiving neoadjuvant therapy, the pathological response was assessed using the tumor regression grade (TRG) according to the Becker classification [17]. Postoperative outcomes included complications classified by the Clavien-Dindo (CD) grading system [18], with severe complications defined as grade ≥ III. Survival data were obtained from hospital records and official registry data, comprising dates of surgery, death, or last known follow-up.

### Clinical management and treatment definitions

Patient management followed standardized institutional protocols based on contemporary German Cancer Society (DKG) guidelines [19]. Preoperative staging routinely involved upper gastrointestinal endoscopy with biopsies, endoscopic ultrasound (EUS), and computed tomography (CT) of the chest and abdomen. All cases were discussed in a multidisciplinary tumor board (MDT) to determine the optimal treatment strategy. Systemic therapy regimens, when indicated, primarily included FLOT (5-fluorouracil [5-FU], leucovorin, oxaliplatin, docetaxel), ECF (epirubicin, cisplatin, 5-FU), or ECX (epirubicin, cisplatin, capecitabine). For the FLOT regimen, therapy typically comprised four preoperative and four postoperative cycles, in accordance with current clinical standards [11]. For ECF/ECX regimens, therapy comprised three preoperative and three postoperative cycles (6 cycles total). Perioperative completion was strictly defined as the administration of the full intended number of neoadjuvant and adjuvant cycles.

For the purpose of this analysis, patients were subsequently categorized into mutually exclusive groups based on the actually received oncological treatment relative to surgery. These categories were: surgery alone (primary TG without chemotherapy), neoadjuvant chemotherapy only (representing the attrition cohort), adjuvant chemotherapy only, and perioperative chemotherapy (completion of both phases).

Total gastrectomy with standardized D2 lymphadenectomy was performed with curative intent. The surgical approach comprised either conventional open surgery or robotic-assisted surgery using the da Vinci^®^ Surgical System (Intuitive Surgical, Sunnyvale, CA, USA). During the study period, the institutional approach transitioned directly from conventional open to robotic-assisted gastrectomy; conventional laparoscopic total gastrectomy was not routinely performed, as robotic assistance was adopted as the primary minimally invasive platform. R0 resection was the primary surgical goal. Pathological specimens were processed according to standard institutional protocols.

Tumor staging was performed according to the respective edition of the UICC TNM classification current at the time of diagnosis/treatment. For patients receiving neoadjuvant therapy, tumor regression was graded using the Becker classification [17]. Postoperative lymph node status was recorded as pN0 (no lymph node metastases) or pN+ (any lymph node metastasis) to facilitate granular nodal burden assessment via the LNR.

### Outcome measures

The primary outcome was 1-year overall survival (OS), defined as the time from the date of surgery to the date of death from any cause or the date of last known contact if alive within the first postoperative year.

To rigorously mitigate immortal time bias — a methodological challenge whereby patients in the treatment-completion group must, by definition, survive long enough to receive adjuvant therapy — a 60-day landmark analysis was conducted. In this analysis, the primary outcome was re-evaluated by excluding all patients who died within the first 60 days following surgery. This 60-day threshold was selected to approximate the typical clinical window required for postoperative recovery and subsequent initiation of the adjuvant phase within the perioperative sequence.

Secondary outcomes encompassed indicators of surgical quality, postoperative morbidity, and short-term mortality. Surgical quality metrics included the R0 resection rate and the median LNR. Postoperative morbidity was captured by the rate of severe complications (Clavien-Dindo ≥ IIIa), including anastomotic leakage and postoperative pneumonia. Additional descriptive endpoints included the reoperation rate, length of hospital stay, 30-day mortality, and in-hospital mortality.

### Statistical analysis

All statistical analyses were performed using IBM SPSS Statistics version 29.0 (IBM Corp., Armonk, NY, USA) and Python version 3.11 with libraries including pandas, numpy, lifelines, tableone, and matplotlib. Baseline patient and tumor characteristics were summarized using descriptive statistics: frequencies and percentages for categorical variables, and median with interquartile range (IQR) or mean with standard deviation (SD) for continuous variables, as appropriate based on data distribution. Overall survival curves were generated using the Kaplan-Meier (KM) method, and survival distributions between groups were compared using the log-rank test.

To identify independent predictors of 1-year OS while avoiding statistical collinearity and the assumption of a linear biological relationship between primary surgery and histopathological response, multivariable Cox proportional hazards regression was performed in two distinct steps. Model 1 evaluated the oncological treatment strategy across the entire cohort (*N* = 145). To isolate the specific prognostic impact of completing the adjuvant phase, this model utilized categorical dummy coding with the “neoadjuvant only” group serving as the reference. Model 2 specifically evaluated the prognostic impact of the pathological TRG exclusively within the subgroup of patients who actually received neoadjuvant chemotherapy (*n* = 85). Within this restricted cohort, TRG was treated as an ordinal variable (Grade 1a = 1, Grade 1b = 2, Grade 2 = 3, Grade 3 = 4) to account for the progressive nature of the histopathological response. Both models included the surgical approach (robotic vs. open) and were adjusted for major clinical confounders: the LNR, preoperative UICC stage, CCI, microscopic resection margin status (R-status), and the occurrence of severe postoperative complications (Clavien-Dindo ≥ III).

To address immortal time bias, a 60-day landmark analysis was performed within the Cox framework, with the time-to-event recalculated from the 60th postoperative day; all early deaths were excluded as previously defined. This landmark sensitivity analysis was applied to both the overall cohort and the neoadjuvant subgroup.

Due to the limited sample size (*n* = 4), the adjuvant chemotherapy only subgroup was excluded from Kaplan-Meier survival analysis to ensure statistical validity. Multicollinearity was assessed using the variance inflation factor (VIF), and the proportional hazards assumption was verified using Schoenfeld residuals. All tests were two-sided, and a p-value < 0.05 was considered statistically significant.

## Results

### Patient and treatment characteristics

Baseline patient demographics and pretreatment clinical features for the primary analysis cohort (*N* = 145) are detailed in Table 1. The cohort had a mean age of 64.3 years with a male predominance (63.4%, *n* = 92). The mean Charlson Comorbidity Index (CCI) was 5.5, indicating substantial baseline comorbidity. Most patients were classified as American Society of Anesthesiologists (ASA) physical status II (49.7%) or III (46.2%).Regarding the actually administered oncological treatment strategy, 41.4% (*n* = 60) underwent surgery alone, 32.4% (*n* = 47) received neoadjuvant chemotherapy only (representing the attrition cohort), and 26.2% (*n* = 38) successfully completed the perioperative chemotherapy sequence. Patients receiving adjuvant chemotherapy alone (*n* = 4) were excluded from this analysis. Among those receiving systemic therapy, FLOT and ECF/ECX were the predominant regimens. Patients completing the perioperative sequence were significantly younger (mean 59.8 vs. 68.0 years, *p* = 0.001) and exhibited fewer comorbidities (mean CCI 4.7 vs. 6.1, *p* = 0.015) compared to the surgery-alone group.

**Table 1:** Baseline patient characteristics (*N* = 145)*

Characteristic	Overall (*N* = 145)	Surgery alone (*n* = 60)	Neoadj. only (*n* = 47)	Perioperative (*n* = 38)	*P*-value
Age (years), mean (SD)	64.3 (12.4)	68.0 (13.9)	63.4 (11.0)	59.8 (10.0)	**0.001**
BMI (kg/m²), mean (SD)	25.6 (5.2)	25.9 (5.5)	25.5 (5.4)	25.2 (4.5)	0.734
Sex (Male / Female), n (%)	92 (63.4) / 53 (36.6)	37 (61.7) / 23 (38.3)	28 (59.6) / 19 (40.4)	27 (71.1) / 11 (28.9)	0.513
ASA-Score (I/II/III/IV), n	5 / 72 / 67 / 1	1 / 31 / 27 / 1	2 / 18 / 27 / 0	2 / 23 / 13 / 0	0.313
Lauren (Intest/Diff/Mix) ^a^, n	68 / 71 / 6	32 / 25 / 3	24 / 23 / 0	12 / 23 / 3	0.147
Pre-op UICC (0/I/II/III/IV) ^b^, n	1 / 43 / 69 / 31 / 1	0 / 36 / 15 / 9 / 0	0 / 6 / 31 / 9 / 1	1 / 1 / 23 / 13 / 0	**< 0.001**
uT-Stage (0/1/2/3/4), n	2 / 20 / 32 / 75 / 16	0 / 17 / 21 / 19 / 3	1 / 3 / 8 / 29 / 6	1 / 0 / 3 / 27 / 7	**< 0.001**
uN-Stage (0/1/2/3), n	58 / 66 / 14 / 7	39 / 13 / 4 / 4	12 / 29 / 4 / 2	7 / 24 / 6 / 1	**< 0.001**
CCI, mean (SD)	5.5 (2.8)	6.1 (3.0)	5.5 (2.9)	4.7 (2.1)	**0.015**
Neo. Regimen (None/ECX, ECF/FLOT), n	60 / 33 / 52	60 / 0 / 0	0 / 16 / 31	0 / 17 / 21	**< 0.001**
Adj. Regimen (None/ECX, ECF/FLOT), n	108 / 20 / 17	60 / 0 / 0	47 / 0 / 0	1 / 20 / 17	**< 0.001**
Neoadjuvant cycles, mean (SD)	2.1 (2.2)	0.0 (0.0)	3.7 (1.5)	3.9 (1.5)	**< 0.001**
Adjuvant cycles, mean (SD)	0.9 (1.7)	0.0 (0.0)	0.0 (0.0)	3.5 (1.6)	**< 0.001**
Total cycles, mean (SD)	2.9 (3.4)	0.0 (0.0)	3.3 (1.9)	6.9 (2.9)	**< 0.001**

*SD* standard deviation, *BMI* body mass index, *ASA* American Society of Anesthesiologists, *CCI* Charlson Comorbidity Index, *UICC* Union for International Cancer Control

* Excluding patients with adjuvant-only therapy (*n* = 4). Bold p-values indicate statistical significance (*p* < 0.05)

^a^ Lauren histological classification

^b^ Preoperative clinical stage based on initial imaging and endoscopy

### Surgical details and postoperative outcomes

Surgical details, postoperative pathological findings, and clinical outcomes are presented in Table 2. Conventional open surgery was performed in 73.1% (*n* = 106) of cases, while robotic-assisted gastrectomy accounted for 26.9% (*n* = 39). The mean operative duration was 5.7 ± 1.8 h across the cohort, with significantly longer procedures observed in the perioperative group (6.2 ± 2.1 h) compared to the surgery-alone group (5.2 ± 1.5 h; *p* = 0.033). Postoperative pathology confirmed pT3 as the most frequent tumor stage (45.5%, *n* = 66), with lymph node metastases (pN+) present in 50.3% of patients (*n* = 73). A microscopically margin-negative resection (R0) was achieved in 95.9% (*n* = 139) of cases. Of the six patients with an R1 resection, all had received neoadjuvant chemotherapy, and the vast majority (83.3%, *n* = 5) subsequently completed the adjuvant phase of the perioperative sequence.

Severe postoperative complications (Clavien-Dindo ≥ III) occurred in 55.9% (*n* = 81) of the cohort, including anastomotic leakage in 15.2% (*n* = 22) and reoperation in 13.1% (*n* = 19). Notably, complication rates did not differ significantly across the treatment groups (*p* = 0.894). In-hospital mortality was 3.4% (*n* = 5). The overall one-year survival rate for the analyzed cohort was 69.0%.

**Table 2:** Surgical details and postoperative outcomes (*N* = 145)

Characteristic / Outcome	Overall (*N* = 145)	Surgery alone (*n* = 60)	Neoadj. only (*n* = 47)	Perioperative (*n* = 38)	*P*-value
Approach (Rob./Open), n (%)	39 (26.9) / 106 (73.1)	10 (16.7) / 50 (83.3)	14 (29.8) / 33 (70.2)	15 (39.5) / 23 (60.5)	**0.040**
Duration of OP (h), mean (SD)	5.7 (1.8)	5.2 (1.5)	5.9 (1.8)	6.2 (2.1)	**0.033**
Blood loss (mL), mean (SD)	356.6 (359.8)	284.0 (291.3)	433.0 (437.2)	376.8 (338.3)	0.238
Harvested LN, mean (SD)	28.6 (11.6)	27.4 (12.0)	29.3 (11.4)	29.6 (11.2)	0.270
Positive LN, mean (SD)	3.5 (5.3)	2.6 (5.2)	4.5 (6.1)	3.4 (4.1)	**0.036**
LNR, mean (SD)	0.1 (0.2)	0.1 (0.2)	0.2 (0.2)	0.1 (0.2)	**0.039**
pT Stage^a^ (0/1/2/3/4), n	8/30/19/66/22	1/25/9/19/6	3/5/4/25/10	4/0/6/22/6	**< 0.001**
pN Stage^b^ (0/1/2/3), n	72/23/19/31	37/8/5/10	19/9/6/13	16/6/8/8	0.244
Post-op UICC (0/I/II/III/IV)^c^, n	10/34/44/43/14	3/27/15/11/4	3/5/16/18/5	4/2/13/14/5	**< 0.001**
Becker TRG (1a/1b/2/3)^d^, n	7/11/48/19	–	3/7/26/11	4/4/22/8	0.844
R-Status (R0/R1)^e^, n (%)	139 (95.9) / 6 (4.1)	60 (100) / 0 (0)	46 (97.9) / 1 (2.1)	33 (86.8) / 5 (13.2)	**0.004**
CD ≥ III (No/Yes)^f^, n (%)	64 (44.1) / 81 (55.9)	26 (43.3) / 34 (56.7)	20 (42.6) / 27 (57.4)	18 (47.4) / 20 (52.6)	0.894
Anast. Leakage (No/Yes), n (%)	123 (84.8) / 22 (15.2)	51 (85.0) / 9 (15.0)	39 (83.0) / 8 (17.0)	33 (86.8) / 5 (13.2)	0.884
Pneumonia (No/Yes), n (%)	133 (91.7) / 12 (8.3)	53 (88.3) / 7 (11.7)	42 (89.4) / 5 (10.6)	38 (100) / 0 (0)	0.096
Re-operation (No/Yes), n (%)	126 (86.9) / 19 (13.1)	52 (86.7) / 8 (13.3)	38 (80.9) / 9 (19.1)	36 (94.7) / 2 (5.3)	0.168
Hospital stay (d), mean (SD)	24.2 (31.4)	28.9 (41.9)	24.5 (26.0)	16.4 (9.8)	**0.010**
ICU stay (d), mean (SD)	8.1 (18.3)	9.8 (20.1)	9.1 (22.2)	4.3 (5.1)	0.129
In-hosp. Mortality (No/Yes), n (%)	140 (96.6) / 5 (3.4)	58 (96.7) / 2 (3.3)	44 (93.6) / 3 (6.4)	38 (100) / 0 (0)	0.276
30-d Mortality (No/Yes), n (%)	142 (97.9) / 3 (2.1)	58 (96.7) / 2 (3.3)	46 (97.9) / 1 (2.1)	38 (100) / 0 (0)	0.528
Relapse (No/Yes), n (%)	130 (89.7) / 11 (7.6)	58 (96.7) / 2 (3.3)	42 (89.4) / 3 (6.4)	30 (78.9) / 6 (15.8)	0.058
Follow-up (d), mean (SD)	1065.5 (1008.5)	1374.8 (1065.8)	666.0 (795.4)	1071.1 (1001.1)	**< 0.001**
1-y Survival (No/Yes), n (%)	45 (31.0) / 100 (69.0)	15 (25.0) / 45 (75.0)	25 (53.2) / 22 (46.8)	5 (13.2) / 33 (86.8)	**< 0.001**

*OP* operation, *LN* lymph node, *LNR* lymph node ratio, *CD* Clavien-Dindo, *TRG* tumor regression grade, *ICU* intensive care unit

Becker TRG applicable only to patients receiving neoadjuvant therapy. Bold p-values indicate statistical significance (*p* < 0.05)

^a^ Pathological tumor stage

^b^ Pathological nodal stage

^c^ Postoperative pathological stage according to UICC TNM classification

^d^ Tumor regression grade according to Becker classification (applicable only to patients receiving neoadjuvant therapy)

^e^ Microscopic resection margin status

^f^ Severe postoperative complications graded according to the Clavien-Dindo classification

### Chemotherapy adherence and attrition analysis

Of the 85 patients who received neoadjuvant chemotherapy, only 38 (44.7%) completed the full perioperative protocol including adjuvant cycles. Conversely, 47 patients (55.3%) discontinued systemic therapy after surgery (neoadjuvant-only group), highlighting substantial attrition in routine clinical practice. Detailed analysis of this attrition cohort (Table 3) revealed that the primary reasons for discontinuing the multimodal sequence were patient refusal or non-compliance (55.3%, 26/47) and medical contraindications or rapid disease progression (34.0%, 16/47). Early postoperative mortality accounted for 10.6% (5/47) of cases.

**Table 3:** Reasons for attrition from adjuvant therapy (*n* = 47)

Reason for Attrition	*n* (%)
Patient Refusal / Non-Compliance	26 (55.3%)
Medical Contraindications / Disease Progression	16 (34.0%)
Early Postoperative Mortality (within 90 days)	5 (10.6%)

### Oncological treatment strategies and survival

**Fig. 2:**
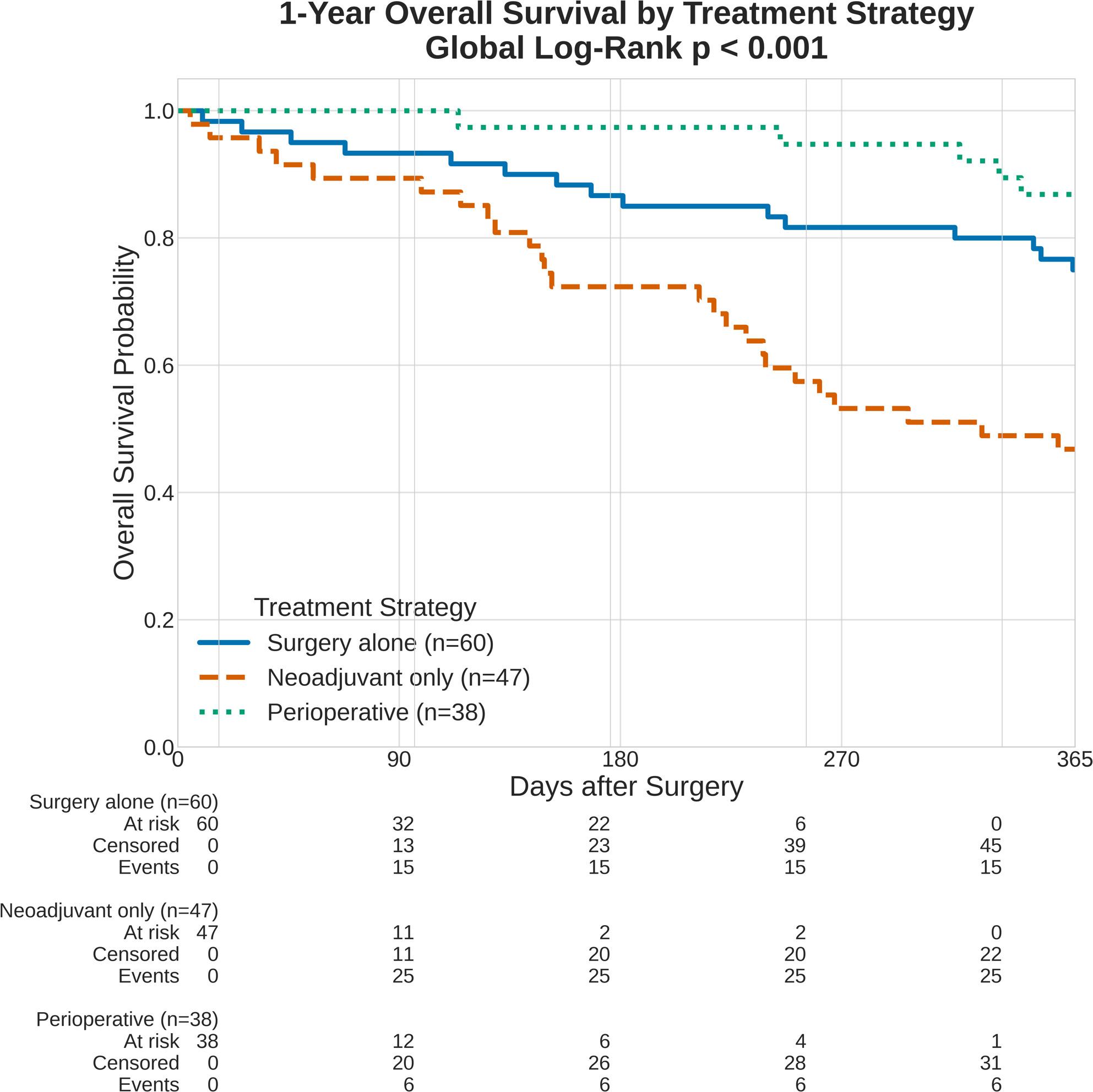
Kaplan-Meier survival curves for oncological treatment strategies. One-year overall survival by treatment group: perioperative completion (n = 38), surgery alone (n = 60), and neoadjuvant only (n = 47). Global log-rank p < 0.001

Kaplan-Meier analysis revealed highly significant differences in one-year OS among the three primary treatment strategy groups (global log-rank *p* < 0.001, Fig. 2). The survival curves demonstrated the highest survival probability for the perioperative chemotherapy group. Conversely, the neoadjuvant chemotherapy-only group exhibited the lowest survival probability of all groups, including the surgery-alone group, which demonstrated intermediate survival.

To rigorously assess independent prognostic factors while avoiding collinearity between primary surgery and histopathological response, multivariable Cox proportional hazards regression was performed in two steps (Table 4). Model 1 evaluated the entire cohort (*N* = 145) using categorical dummy coding with the neoadjuvant-only group as the reference. This model confirmed a strong, independent association between perioperative completion and improved one-year OS (HR = 0.20, 95% CI 0.08–0.50, *p* = 0.001). Furthermore, baseline comorbidity significantly impacted early survival, with each unit increase in the CCI associated with an 11% increase in the hazard of mortality (HR = 1.11, 95% CI 1.02–1.22, *p* = 0.022).

**Table 4:** Hazard ratios and 95% confidence intervals from multivariable cox regression analysis (*N* = 145)*

Covariate	Hazard Ratio (HR)	95% CI (Lower – Upper)	*P*-value
Model 1: Overall Cohort (*N* = 145)			
Treatment Strategy (Ref: Neoadj. only)			
– Perioperative completion	0.20	0.08–0.50	**0.001**
– Surgery alone	0.36	0.18–0.73	**0.004**
Surgical Approach (Robotic vs. Open)	0.88	0.41–1.89	0.745
Resection Margin Status (R1 vs. R0)	3.05	0.75–12.38	0.118
Lymph Node Ratio (LNR)	1.63	0.35–7.61	0.534
Severe Complications (CD ≥ III)	1.67	0.86–3.23	0.130
Preoperative UICC Stage	0.97	0.60–1.58	0.903
Charlson Comorbidity Index (CCI)	1.11	1.02–1.22	**0.022**
Model 2: Neoadjuvant Subgroup (*n* = 85)			
Becker TRG (Ordinal, 1–4)	1.60	0.98–2.59	0.059
Periop. completion (vs. Neoadj. only)	0.18	0.07–0.48	**0.001**
Surgical Approach (Robotic vs. Open)	1.00	0.41–2.47	0.996
Resection Margin Status (R1 vs. R0)	5.36	1.15–25.00	**0.033**
Lymph Node Ratio (LNR)	0.58	0.06–5.86	0.645
Severe Complications (CD ≥ III)	1.49	0.67–3.31	0.333
Preoperative UICC Stage	0.47	0.23–0.96	**0.038**
Charlson Comorbidity Index (CCI)	1.19	1.04–1.37	**0.010**

*CI* confidence interval, *CD* Clavien-Dindo classification, *CCI* Charlson Comorbidity Index

* To avoid collinearity, multivariable analysis was performed in two steps. Model 1 evaluates treatment strategies across the entire cohort (*N* = 145). Model 2 evaluates TRG exclusively within the neoadjuvant-treated subgroup (*n* = 85). Bold values indicate statistical significance (*p* < 0.05)

TRG: ordinal variable (1 = Grade 1a, 2 = Grade 1b, 3 = Grade 2, 4 = Grade 3). LNR: continuous variable. Surgical approach: robotic vs. open (reference)

### Landmark sensitivity analysis for immortal time bias

To address the potential for immortal time bias — whereby patients in the perioperative group inherently survived long enough to receive adjuvant therapy — a 60-day landmark sensitivity analysis was conducted (Supplementary Table 1). After shifting the time-to-event baseline to 60 days post-surgery and excluding early deaths (*N* = 137), the analysis confirmed that the survival advantage associated with perioperative completion remained robust and statistically significant (HR = 0.23, 95% CI 0.09–0.62, *p* = 0.004). The corresponding Kaplan-Meier survival curves for the landmark cohort are shown in Supplementary Fig. 1.

Likewise, this protective effect persisted within the neoadjuvant-treated landmark subgroup (HR = 0.21, *p* = 0.003). This finding validates that the superior early survival associated with completing multimodal therapy represents a genuine oncological effect rather than an artifact of early surgical mortality.

### Tumor regression grade and survival

**Fig. 3:**
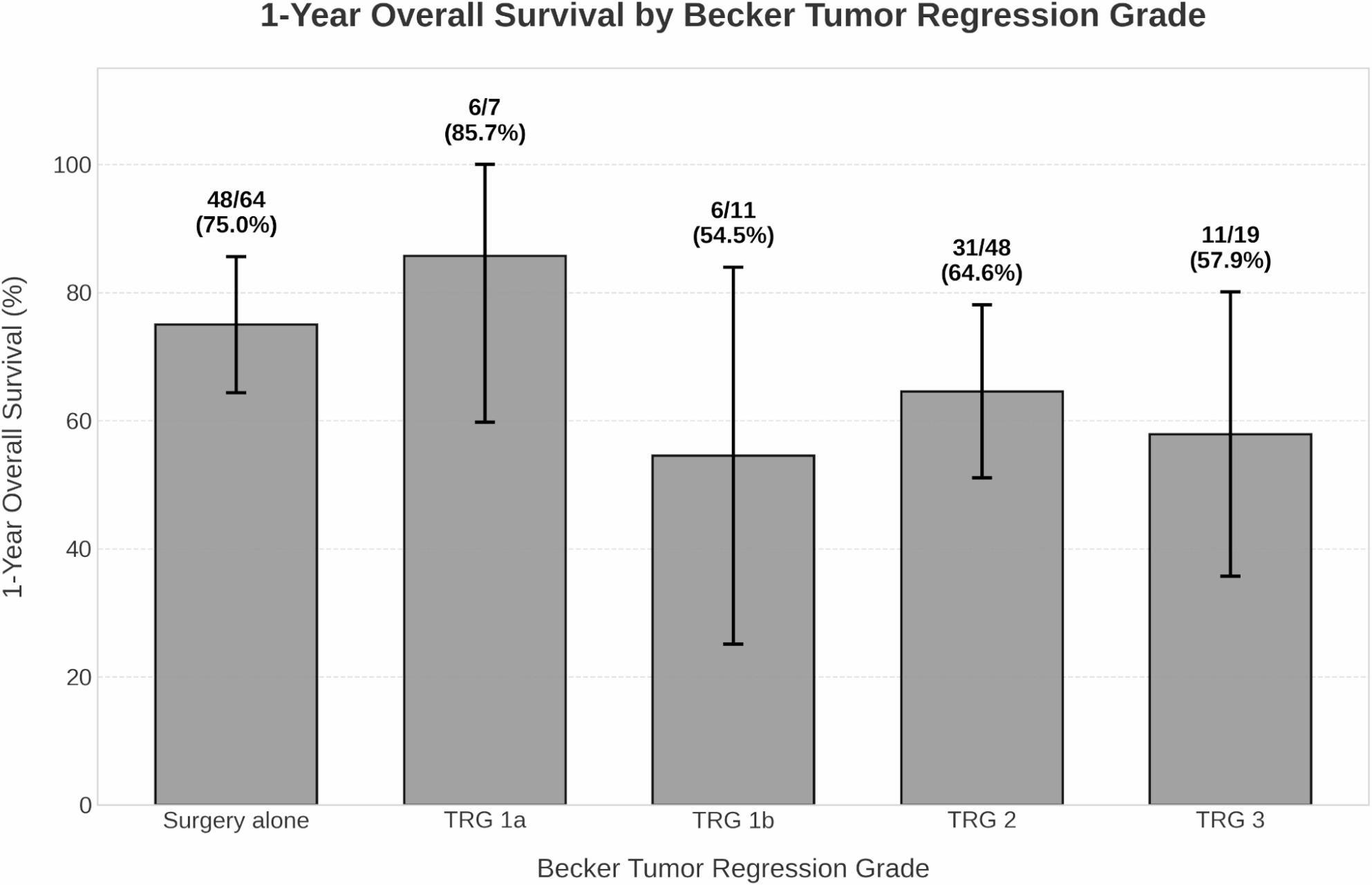
One-year overall survival rates by Tumor Regression Grade (TRG, Becker classification). The surgery-alone group is included as a comparator without neoadjuvant therapy

Pathological tumor regression grade (TRG) according to the Becker classification was assessed in the 85 patients who received neoadjuvant chemotherapy. One-year survival rates varied notably by TRG category: the highest survival was observed for TRG 1a (85.7%), followed by the surgery-alone group as a comparator (75.0%), TRG 2 (64.6%), TRG 3 (57.9%), and TRG 1b (54.5%) (Fig. 3). To isolate the prognostic impact of TRG from patients who did not receive systemic therapy, multivariable Model 2 evaluated TRG exclusively within the neoadjuvant-treated subgroup (*n* = 85, Table 4). In this restricted cohort, ordinal TRG analysis revealed a prognostic trend toward increased mortality that did not reach formal statistical significance with poorer response grades (HR = 1.60, 95% CI 0.98–2.59, *p* = 0.059). Furthermore, within this chemotherapy-treated cohort, an R1 resection margin emerged as a highly significant independent predictor of mortality (HR = 5.36, *p* = 0.033). Subgroup analysis (Supplementary Table 2) uncovered a notable clinical paradox: although the TRG 1b cohort demonstrated excellent primary tumor regression, this subgroup concurrently exhibited the highest baseline comorbidity burden (mean CCI 7.00), the highest rate of R1 resections (18.2%), and the highest mean LNR (0.19), findings that correlate with their markedly lower observed survival rate (54.5%).

### Lymph node ratio (LNR) and survival

**Fig. 4:**
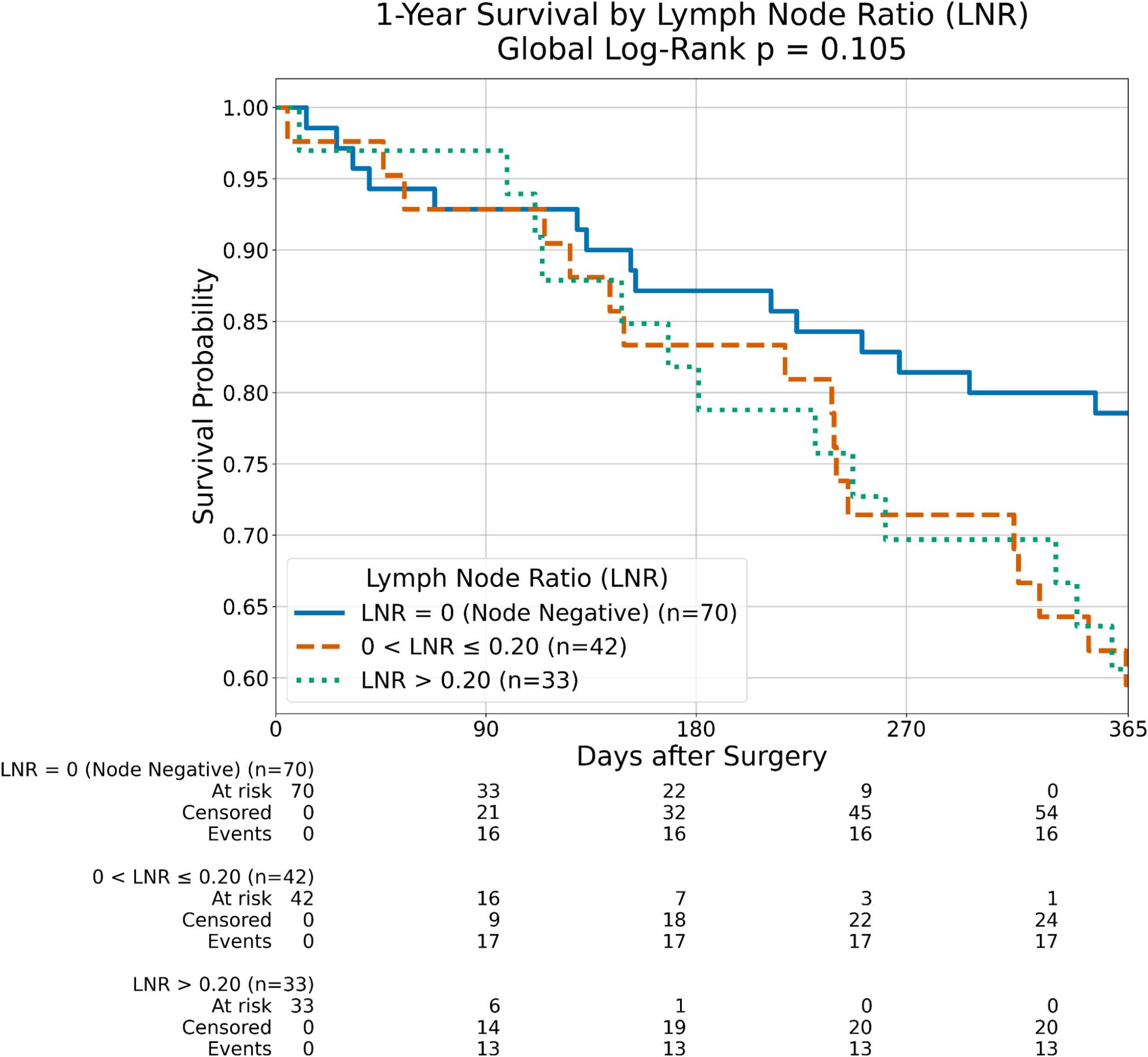
Kaplan-Meier survival curves for Lymph Node Ratio (LNR). One-year overall survival stratified by LNR = 0 (node negative, n = 70), 0 < LNR ≤ 0.20 (n = 42), and LNR > 0.20 (n = 33). Global log-rank p = 0.105

To provide a more granular assessment of nodal disease burden, the LNR was employed instead of binary pN status. While Kaplan-Meier analysis indicated a trend toward improved one-year survival for node-negative patients (LNR = 0) compared to those with an elevated LNR (log-rank *p* = 0.105, Fig. 4), this difference did not retain independent significance in the adjusted multivariable model (Model 1: HR = 1.63, 95% CI 0.35–7.61, *p* = 0.534).

### Surgical approach and complications

Kaplan-Meier analysis revealed comparable one-year survival probabilities between patients undergoing robotic-assisted total gastrectomy (*n* = 39) and conventional open total gastrectomy (*n* = 106, log-rank p = n.s.). This finding was further corroborated by the multivariable Cox regression analysis (HR = 0.88, 95% CI 0.41–1.89, *p* = 0.745). Furthermore, while the occurrence of severe postoperative complications (Clavien-Dindo ≥ III) was prevalent across the cohort, their inclusion in the multivariable model demonstrated a trend toward increased mortality that did not reach statistical significance (HR = 1.67, 95% CI 0.86–3.23, *p* = 0.130) and did not diminish the strong, independent survival benefit associated with completing the perioperative treatment sequence.

Notably, in the 60-day landmark analysis (Supplementary Table 1), the predictive value of severe complications diminished further (HR = 1.31, *p* = 0.450), suggesting that for patients who survive the initial 60-day postoperative recovery phase, long-term prognosis is dictated primarily by tumor biology and systemic treatment adherence rather than by initial surgical morbidity.

## Discussion

This study investigated prognostic factors influencing 1-year overall survival following curative-intent TG for gastric cancer, with particular attention to real-world clinical confounders and methodological biases. A central finding is the pronounced, independent survival benefit associated with completing the full perioperative chemotherapy sequence. Consistent with the established paradigm of multimodal therapy [7, 20], our data provide strong evidence for the biological efficacy of the adjuvant phase.

However, maintaining treatment continuity remains a substantial challenge in routine clinical practice. Comparative data from the landmark FLOT4 trial [11] demonstrated that even under controlled study conditions, only 60% of patients initiated the adjuvant phase, and merely 46% completed the full protocol. Our real-world findings align closely with these figures but reveal an even more pronounced attrition rate: among the 85 patients who initiated neoadjuvant therapy, 55.3% (*n* = 47) failed to proceed to adjuvant treatment, yielding a completion rate of only 44.7% (*n* = 38). Detailed analysis of our attrition cohort provides valuable clinical context frequently absent from retrospective analyses. The primary drivers for discontinuing therapy were patient refusal or non-compliance (55.3%) and medical contraindications or physical exhaustion (34.0%). Early postoperative mortality accounted for only a minor fraction (10.6%) of attrition. These findings underscore the importance of completing the full perioperative course when feasible, rather than focusing solely on the neoadjuvant component, and highlight the critical need for careful patient selection and individualized treatment approaches [21].

Addressing a central methodological concern in the evaluation of perioperative treatment pathways, we rigorously mitigated the risk of immortal time bias [22]. This bias arises because patients in the perioperative completion cohort must, by definition, survive the postoperative period to receive adjuvant therapy, which can artificially inflate their early survival curves compared to those who discontinue treatment early. By conducting a 60-day landmark sensitivity analysis and excluding all early postoperative deaths, we demonstrated that the significant survival divergence between the completion and attrition cohorts persisted (HR = 0.23, *p* = 0.004). This robustly confirms that the superior survival of the perioperative group is not merely an artifact of survivorship bias but reflects a genuine oncological advantage of completing the multimodal sequence. Nevertheless, residual selection bias cannot be entirely excluded, as patients who completed the perioperative sequence were significantly younger and had fewer comorbidities, factors that may have independently contributed to their favorable outcomes beyond the effect of treatment completion itself.

Furthermore, addressing a critical determinant of oncological outcome, we integrated the microscopic resection margin (R-status) into our multivariable models. R-status can function as a major confounding variable in retrospective survival analyses, potentially skewing the perceived efficacy of systemic treatments if not adequately controlled for [23]. In our cohort, incomplete resection (R1) was infrequent (*n* = 6 vs. *n* = 139 for R0). However, when restricting the analysis to patients who received neoadjuvant chemotherapy to assess the biological response, an R1 margin emerged as a highly significant independent predictor of mortality (HR = 5.36, *p* = 0.033), an effect that was further amplified within the landmark subgroup surviving beyond 60 days (HR = 6.27, *p* = 0.029). Clinical profiling of these R1 patients revealed aggressive underlying tumor biology: compared to the R0 cohort, R1 patients exhibited a substantially higher nodal burden (mean LNR 0.258 vs. 0.121), poorer histopathological response (mean TRG 2.83 vs. 1.62), and consequently inferior 1-year OS (50.0% vs. 69.2%). This aggressive biology was directly reflected in their postoperative trajectories: nearly all patients (*n* = 5) experienced rapid disease progression (including 2 cases of peritoneal carcinomatosis), and only 1 patient remained disease-free. Paradoxically, despite the absence of severe surgical complications (0%) and a notably high rate of adjuvant therapy completion (83.3%), their survival remained severely compromised by residual tumor burden. By adjusting for margin status, we demonstrate that the pronounced survival benefit of completing the perioperative sequence constitutes a genuine, independent biological advantage, rather than an artifact attributable to varying surgical radicality. However, these R1 findings should be interpreted with caution given the small number of R1 resections (*n* = 6), which limits statistical power and precludes definitive conclusions.

Our study also confirmed the established role of key pathological factors in determining prognosis after TG. Following recent methodological recommendations, we utilized the LNR rather than binary pN status to provide a more granular assessment of nodal disease burden [24]. While increasing LNR demonstrated a trend toward poorer 1-year survival in univariable analysis, it did not emerge as an independent predictor in our multivariable Cox model (HR = 1.63, *p* = 0.534). This finding suggests that in the context of multimodal therapy, early survival dynamics may be governed more heavily by systemic treatment response and baseline patient physiology than by nodal status alone, provided that adequate oncological resection, including systematic D2 lymphadenectomy, is performed [7]. Indeed, extensive D2 lymphadenectomy has been shown to effectively neutralize the adverse prognostic impact of occult nodal micrometastases [25], further explaining why granular nodal indices such as LNR may lose their independent prognostic significance in a well-resected, multimodally treated cohort.

Similarly, the pathological TRG after neoadjuvant chemotherapy emerged as a notable prognostic factor in our cohort, consistent with previous reports [26, 27]. A favorable TRG correlated with improved survival prospects, likely reflecting higher chemosensitivity and less aggressive disease. By methodologically restricting our analysis to the subgroup of patients who actually received neoadjuvant chemotherapy, we eliminated the biological confounding introduced by primary surgery cases. Within this refined model, treating TRG as an ordinal variable confirmed a prognostic trend demonstrating a progressive increase in mortality risk with poorer histopathological response (HR = 1.60, *p* = 0.059). Of particular clinical interest, our subgroup analysis revealed a highly relevant nuance regarding the TRG 1b cohort. Despite demonstrating excellent primary tumor regression, this subgroup exhibited the highest rates of positive resection margins (18.2% R1) and the highest mean LNR (0.194), which directly correlated with a pronounced reduction in early survival. This phenomenon suggests that in a subset of patients, the primary tumor and locoregional lymph nodes may exhibit divergent sensitivities to systemic therapy, underscoring the importance of comprehensive postoperative risk stratification [13]. Integrating TRG with LNR and molecular markers (e.g., MSI, HER2) may further enhance prognostic precision [28, 29].

Regarding the surgical approach, our analysis detected no statistically significant difference in 1-year overall survival between patients undergoing robotic-assisted versus conventional open total gastrectomy (HR = 0.88, *p* = 0.745). This finding is consistent with multiple previous studies and meta-analyses reporting comparable short-term and mid-term oncological outcomes between minimally invasive and open gastrectomy for gastric cancer, provided that established oncological principles including R0 resection and adequate lymphadenectomy are strictly adhered to [30, 31].

While potential benefits of the robotic approach with respect to reduced intraoperative blood loss or shorter hospital stays have been reported [32, 33], our results indicate that, regarding early survival, adherence to oncological surgical standards remains paramount. The choice between robotic and open surgery should therefore integrate considerations of oncological safety with patient factors, surgeon expertise, and institutional capabilities [34, 35].

In addition to oncological outcomes, perioperative morbidity remains a critical consideration. In our cohort, the rate of severe complications (Clavien-Dindo ≥ III) (55.9%), the reoperation rate (13.1%), and anastomotic leakage (15.2%) were higher than those reported in recent benchmarks [36]. These rates likely reflect the complexity and risk profile of patients treated at our tertiary center, characterized by a high mean CCI (5.5) and a predominance of advanced stages (cT3/4 in > 65%), representing a high-risk real-world population often underrepresented in controlled trials. To address this rigorously, we included both the CCI and severe postoperative complications (Clavien-Dindo ≥ III) as adjusting covariates in our multivariable Cox model. Notably, upon applying the 60-day landmark analysis, severe complications lost their predictive association with mortality (HR = 1.31, *p* = 0.450). This observation suggests that while severe complications strongly impact early in-hospital mortality, patients who survive the initial 60-day recovery phase face a prognosis dictated primarily by tumor biology and chemotherapy completion, rather than by antecedent surgical adverse events.

### Study limitations

This study has several limitations. First, its retrospective, single-center design may introduce selection bias and unmeasured confounders, potentially limiting the generalizability of the findings. Furthermore, the restriction to total gastrectomy, while ensuring cohort homogeneity regarding surgical morbidity, limits the applicability of these results to patients undergoing subtotal or distal gastrectomy. While retrospective comparisons of multimodal treatment sequences are inherently prone to immortal time bias, we proactively mitigated this through a 60-day landmark analysis. Second, although we successfully integrated severe postoperative complications and R-status into our multivariable Cox models to control for surgical morbidity and resection quality, the absolute number of patients in certain subgroups (e.g., R1 resections, *n* = 6) was small. Furthermore, patients receiving adjuvant chemotherapy only (*n* = 4) were excluded from the primary survival cohort (*N* = 145) to maintain methodological robustness.

Third, the one-year follow-up period is insufficient to evaluate long-term survival and late recurrences. Our study specifically addresses early survival dynamics and the immediate prognostic impact of treatment adherence; however, long-term outcomes warrant investigation in future analyses. Finally, the absence of comprehensive molecular and genetic profiling (e.g., microsatellite instability [MSI], human epidermal growth factor receptor 2 [HER2] status) limits insights into individual tumor biology, which undoubtedly influences treatment outcomes. Given the overall sample size, larger prospective multicenter studies are warranted to confirm these findings and further refine personalized perioperative strategies.

## Conclusions

This study demonstrates the profound, independent prognostic value of completing the full perioperative chemotherapy sequence in patients undergoing curative total gastrectomy for gastric cancer. Supported by a robust landmark analysis that mitigated immortal time bias, our findings establish that this survival benefit represents a genuine oncological advantage rather than an artifact of early postoperative mortality. Patients who failed to proceed to adjuvant therapy demonstrated significantly worse one-year survival. The substantial attrition rate of 55.3% in this real-world cohort identifies treatment completion as a major clinical bottleneck, primarily driven by patient refusal and medical contraindications.

Moreover, when evaluated specifically within the neoadjuvant-treated cohort, a poor histopathological response (higher TRG) demonstrated a prognostic trend toward early mortality, although this did not reach formal statistical significance (*p* = 0.059), underscoring the potential role of primary tumor chemosensitivity. The surgical approach — robotic versus open — exerted no measurable impact on early survival. Importantly, while high baseline comorbidities and positive resection margins (R1) affected clinical trajectories, the overarching oncological advantage of completing the multimodal sequence remained statistically robust after adjusting for these confounders. These findings support the prioritization of complete perioperative treatment and careful risk stratification. Future efforts should aim to optimize patient prehabilitation, improve adherence to adjuvant therapies, and incorporate molecular profiling to personalize and improve long-term survival strategies.

## Supplementary Material

Supplementary Material 1.

## Data Availability

The datasets used and/or analysed during the current study are available from the corresponding author on reasonable request.
