# Supplementary material for: Completion of perioperative chemotherapy and tumor regression grade as independent predictors of one-year survival after total gastrectomy for gastric cancer: a retrospective cohort study
Source: World J Surg Oncol. 2026 Apr 25;24:189. doi: 10.1186/s12957-026-04364-w (PMC13109887; doi:10.1186/s12957-026-04364-w)

**Supplementary Table 1: Sensitivity Analysis – 60-Day Landmark Multivariable Cox Regression (N = 145)***

| **Covariate** | **Hazard Ratio (HR)** | **95% CI (Lower – Upper)** | **P-value** |
| --- | --- | --- | --- |
| ***Model 3: Overall Cohort Landmark (N = 137)*** |  |  |  |
| **Treatment Strategy (Ref: Neoadj. only)** |  |  |  |
| – Perioperative completion | 0.23 | 0.09 – 0.62 | **0.004** |
| – Surgery alone | 0.33 | 0.15 – 0.73 | **0.006** |
| Surgical Approach (Robotic vs. Open) | 0.96 | 0.42 – 2.22 | 0.925 |
| Resection Margin Status (R1 vs. R0) | 3.21 | 0.75 – 13.79 | 0.116 |
| Lymph Node Ratio (LNR) | 2.82 | 0.55 – 14.44 | 0.213 |
| Severe Complications (CD ≥ III) | 1.31 | 0.65 – 2.67 | 0.450 |
| Preoperative UICC Stage | 0.79 | 0.45 – 1.40 | 0.415 |
| Charlson Comorbidity Index (CCI) | 1.09 | 0.99 – 1.21 | 0.093 |
| ***Model 4: Neoadjuvant Subgroup Landmark (n = 80)*** |  |  |  |
| Becker TRG (Ordinal, 1–4) | 1.53 | 0.91 – 2.57 | 0.106 |
| Periop. completion (vs. Neoadj. only) | 0.21 | 0.08 – 0.59 | **0.003** |
| Surgical Approach (Robotic vs. Open) | 1.25 | 0.46 – 3.44 | 0.664 |
| Resection Margin Status (R1 vs. R0) | 6.27 | 1.21 – 32.42 | **0.029** |
| Lymph Node Ratio (LNR) | 1.08 | 0.10 – 11.55 | 0.947 |
| Severe Complications (CD ≥ III) | 1.21 | 0.51 – 2.89 | 0.667 |
| Preoperative UICC Stage | 0.35 | 0.15 – 0.78 | **0.011** |
| Charlson Comorbidity Index (CCI) | 1.17 | 1.01 – 1.35 | **0.034** |

** 60-day landmark multivariable Cox proportional hazards regression. Patients who died within 60 days of surgery were excluded to mitigate immortal time bias. Model 3 evaluates the entire landmark cohort (N = 137). Model 4 restricts the analysis to landmark patients who received neoadjuvant therapy (n = 80).*

*Bold values indicate statistical significance (p < 0.05).*

*TRG: ordinal variable (1 = Grade 1a, 2 = Grade 1b, 3 = Grade 2, 4 = Grade 3). LNR: continuous variable.*

*Abbreviations: CI, confidence interval; CD, Clavien-Dindo classification; CCI, Charlson Comorbidity Index*

**Supplementary Table 2: Comprehensive Subgroup Analysis by Tumor Regression Grade (TRG) vs. Surgery Alone**

| **Subgroup Characteristics** | **Surgery alone (n = 60)** | **TRG 1a (n = 7)** | **TRG 1b (n = 11)** | **TRG 2 (n = 48)** | **TRG 3 (n = 19)** |
| --- | --- | --- | --- | --- | --- |
| 1-Year OS, n/N (%) | 45/60 (75.0%) | 6/7 (85.7%) | 6/11 (54.5%) | 31/48 (64.6%) | 11/19 (57.9%) |
| Age, mean (years) | 68.0 | 58.5 | 61.2 | 62.0 | 62.8 |
| ASA Score, mean | 2.4 | 2.29 | 2.55 | 2.33 | 2.63 |
| CCI, mean | 6.1 | 3.71 | 7.00 | 5.08 | 4.74 |
| Pre-op UICC stage, mean | 1.6 | 1.71 | 2.09 | 2.21 | 2.32 |
| LNR, mean | 0.10 | 0.00 | 0.19 | 0.16 | 0.13 |
| R1 Resection, n/N (%) | 0/60 (0.0%) | 0/7 (0.0%) | 2/11 (18.2%) | 3/48 (6.2%) | 1/19 (5.3%) |
| CD ≥ III, n/N (%) | 34/60 (56.7%) | 4/7 (57.1%) | 4/11 (36.4%) | 30/48 (62.5%) | 9/19 (47.4%) |

*TRG according to Becker classification. Surgery alone group included as comparator (no neoadjuvant therapy received).*

*Abbreviations: OS, overall survival; ASA, American Society of Anesthesiologists; CCI, Charlson Comorbidity Index; UICC, Union for International Cancer Control; LNR, lymph node ratio; CD, Clavien-Dindo classification.*

**Supplementary Figure 1: Kaplan-Meier survival curves for the 60-day landmark cohort by treatment strategy (N = 137). Patients who died within 60 days of surgery were excluded to mitigate immortal time bias. Global log-rank p < 0.001.**


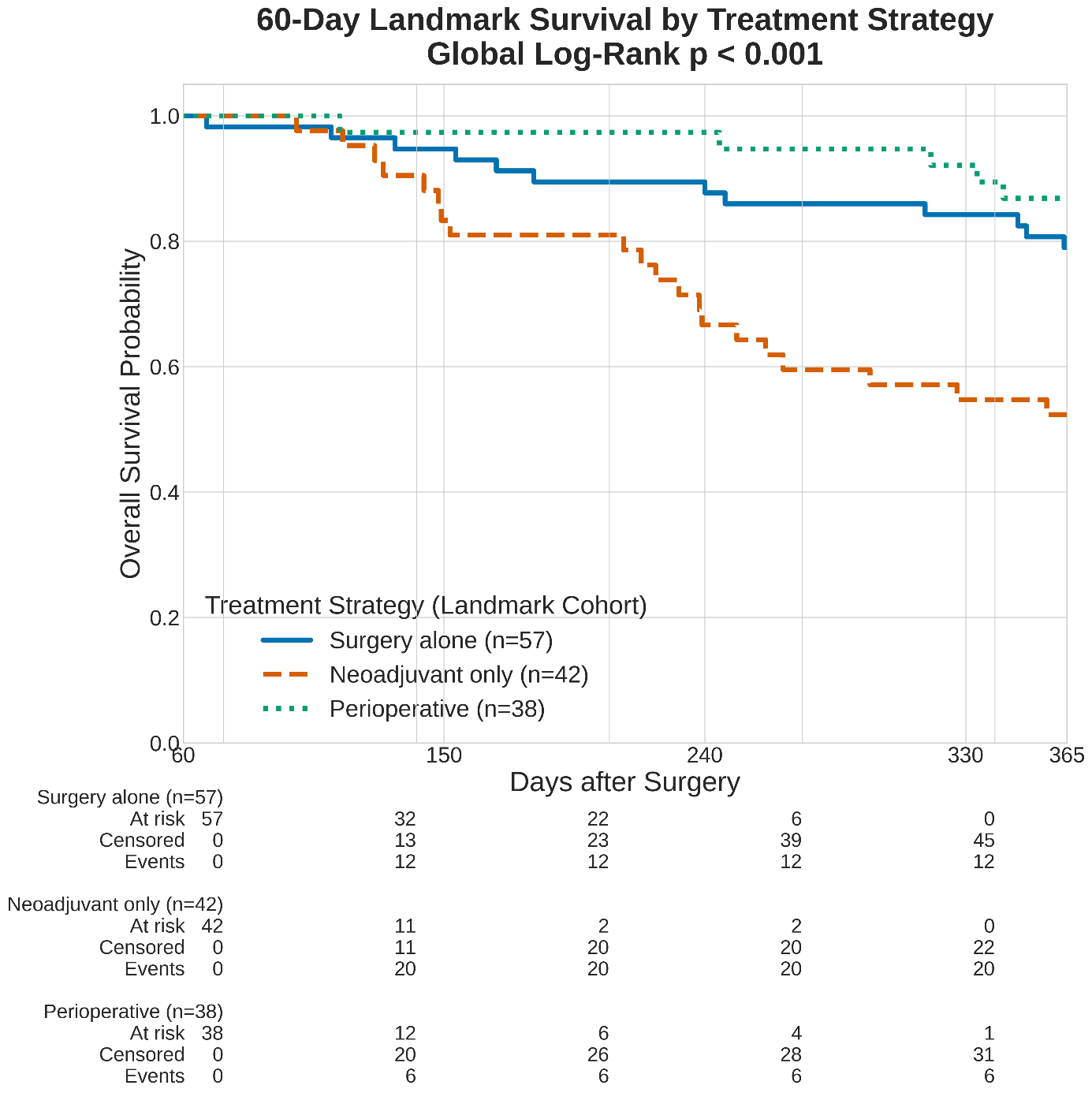

Supplement: Supplementary file 1 — Supplementary Material 1. [file 12957_2026_4364_MOESM1_ESM.docx]
